# Dynamic Control Scheme of Multiswarm Persistent Surveillance in a Changing Environment

**DOI:** 10.1155/2019/6025657

**Published:** 2019-01-16

**Authors:** Tian Jing, Weiping Wang, Tao Wang, Xiaobo Li, Xin Zhou

**Affiliations:** College of Systems Engineering, National University of Defense Technology, Changsha 410073, China

## Abstract

The persistent surveillance problem has been proved to be an NP hard problem for multiple unmanned aerial vehicle systems (UAVs). However, most studies in multiple UAV control focus on control cooperative path planning in a single swarm, while dynamic deployment of a multiswarm system is neglected. This paper proposes a collective control scheme to drive a multiswarm UAVs system to spread out over a time-sensible environment to provide persistent adaptive sensor coverage in event-related surveillance scenarios. We design the digital turf model to approximate the mixture information of mission requirements and surveillance reward. Moreover, we design a data clustering-based algorithm for the dynamic assignment of UAV swarms, which can promote workload balance, while also allowing real-time response to emergencies. Finally, we evaluate the proposed architecture by means of simulation and find that our method is superior to the conventional control strategy in terms of detection efficiency and subswarm equilibrium degree.

## 1. Introduction

The control strategy of multiple unmanned aerial vehicles (UAVs) for remote-sensing applications such as search, surveillance, and patrol has been a topic of rising interest over the last several years [1–3]. Compared with single UAVs, multiple UAVs with advanced control mechanisms have many advantages as they are more robust and can synchronize multiple tasks.

There have been many studies for the one-time surveillance task [4]. The main purpose of these researches is the minimization of the UAV swarm time to find the most targets, maximization of the target detection probability, or maximization of the coverage area [5].

However, in many cases, the mission environment changes continually, and emergencies occur over time. Such missions often require UAVs to fly over the target area continually, which is called persistent surveillance [6]. Persistent surveillance applications include crowd monitoring in open areas, border patrolling, climate reconnaissance, and wilderness search and rescue (WiSAR) [7–10]. Moreover, emergency situations complicate matters; for example, in an earthquake search and rescue scenario, unexpected aftershocks may occur at any time and the surveillance system is required to respond as fast as possible.

Persistent surveillance problems are normally used to describe scenarios wherein a certain area or group of targets needs to be closely observed. In persistent surveillance problems, the main task is to design a control mechanism to maintain the performance of the surveillance over the whole mission period. In traditional area coverage methods, “persistent” refers to a long period, and not to the infinite continuity in the time domain. Owing to the progress in energy management of UAVs, UAVs are now able to conduct a 24/7 persistent mission [11, 12]. However, the method of precisely describing the mission environment to update the environment and mission order remains an urgent problem. In particular, the strategy and scheme adopted in this study make a considerable difference to the problem of deploying a multiswarm UAV system to gather information in a changing environment.

In natural ecological environments, the growth of vegetation in turf shares some common features with the refreshing of information in persistent surveillance missions. Both vegetation and information grow over time and converge to their maximum limits while their growth rate and limits vary with time and space. Moreover, the aim of UAV swarms in persistent surveillance missions is to gather more information; this has many similarities to the aim of herbivorous animals in pursuit of turf.

Inspired by nature, we present a distributed digital turf model (DTM) to describe the updating process of persistent surveillance. Refreshing of information in the environment and the changes in mission overlap in the DTM map. To automatically set the deployment and configuration of UAV swarms, a cluster-based control scheme is used on the basis of the proposed DTM map. The main contributions of our proposed method are listed as follows.

We investigate the problem of multiswarm UAV surveillance and provide a two-layer collective control scheme for the deployment of UAV swarms and the number of UAVs in each swarm. Our methods decouple the resource allocation and subregion decomposition from motion planning.

We propose the digital turf model (DTM) as a spatiotemporal field to approximate the time-sensible target space and mission request. The interaction of UAVs and targets is simulated in the model.

We design a data clustering-based algorithm for the motion plan of the UAV swarms, and the resource allocation of the system between swarms is processed based on the DTM weight of the subregions.

The rest of the paper is organized as follows: Section 2 discusses related work on persistent surveillance using swarming UAVs. The problem framework, environment model, and UAV swarm model are given in Section 3.  Section 4 is devoted to the control scheme of multiswarms, and the proposed method is discussed in detail.  Section 5 presents the simulation results of the proposed method. Finally, we present our conclusions and discuss possible future work in Section 6.

## 2. Related Works

Multi-UAV searching has always been a primary problem in multi-UAV cooperation theory, and its extension to persistent surveillance presents greater challenges. There has been a rising interest in multi-UAV persistent surveillance as a result of emerging demands of real-time continuous information gathering.

With the improvement in UAVs conducting 24/7 persistent missions and the growing demands of real-time continuous information of the environment, the multi-UAV persistent surveillance task has been studied in many research studies.

The persistent surveillance of single UAVs has been studied extensively, with typical searching methods including optimization such as spanning tree coverage [13], dynamic programming [14], area partition [15], potential field methods [16], and other swarming approaches [17, 18].

The discrete environment model is mostly used in the path planning process for single-UAV persistent surveillance, turning the problem into classical search problems such as the traveling salesperson problem [19]. Several methods have been used for environment decomposition, such as approximate cellular decomposition, constrained Delaunay triangulation (CDT), rectilinear partition, and identical regular hexagonal cells [20–22]. The states of grids in the environment can be described using the dual-value matrix or finite-state machine (FSM) [23]. Meanwhile, tools such as the Markov chain are used to model the target with discrete states [24]. The environment model with nondiscrete target states is less common. Some of the methods include refresh time, which is the time elapsed since it was last observed, scalar field [25], and entropy [26].

The searching problem is already an NP hard problem for a single agent, and the problem is further complicated with multiple agents with an infinite time [27]. Sensor networks are presented to observe the target area continuously, and distributed and multilevel control methods are used for the configuration of sensors [28, 29]. Meanwhile, the coordinate persistent surveillance in the complex environment with dynamics with large UAV swarms brings forth a whole new suite of problems associated with environment evaluation, communication constraints, and balance of task load between swarms. In confront with multiple UAVs, collective control architecture, resource allocation, and motion coordination must be taken into consideration.

Thus far, many researchers have investigated collaborative mechanisms to control multiple UAVs. One common way to simplify the problem is by workspace partition. The environment is divided into optimal subregions, so that each agent only need to consider the coverage of its own region. Voronoi partition is often used to divide the workspace while maintaining workload balance [30]. Moreover, dynamic replanning of Voronoi diagrams is used for persistent task [31]. K-means clustering is also a very effective method in data science which can promote the workload balance in subregions [32].

## 3. Problem Formulation

### 3.1. Environment Model

**Figure 1:**
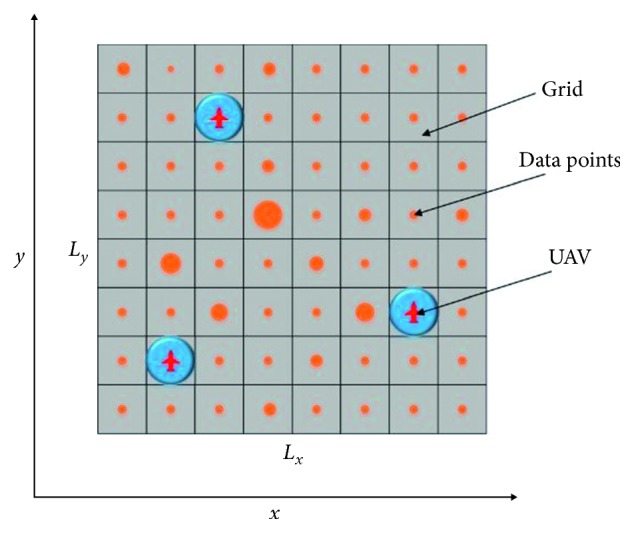
Region decomposition.

In this paper, we assume that all of the UAVs in the swarm conduct the persistent surveillance mission over a fixed area *E* ⊂ *ℝ*^2^ at a constant height. While the UAVs move in a continuous space, we use a discrete method to represent the environment state. As shown in Figure 1, the target space is decomposed into separate grids using an *approximate cellular decomposition*—where the footprint of the UAVs equals the size of the grid:

(1) $$\begin{array}{c} E=L_{x}\times L_{y}. \end{array}$$

In addition, it is assumed that the field of view (FOV) can cover the grid exhaustively. The area of interest (AOI) in the mission environment is a set of time-sensible grids *Q*(*t*) ⊂ *E*, where the total number of AOI grids *N*_AOI_(*t*) can be defined as follows:

(2) $$\begin{array}{c} N_{\text{AOI}}\left(t\right)=\overset{L_{x}}{\underset{i=1}{\sum }}\overset{L_{y}}{\underset{j=1}{\sum }}p_{ij}\;\text{for}\;\;p_{ij}\subset Q\left(t\right), \end{array}$$

where *p*_*ij*_(*i* ∈ {1,2,…, *L*_*x*_}, *j* ∈ {1,2,…, *L*_*y*_}) stands for a grid in the environment, and each grid in an AOI has an associated information reward value. We model the reward with a spatiotemporal field Φ(*t*)=*Q*(*t*) × *ℝ*^2^, and the aim of the surveillance is to minimize Φ(*t*).

### 3.2. UAV Swarm Model

The UAVs surveillance system consists of multiple UAV swarms that each move independently along a trajectory and conduct their own surveillance mission.

As discussed in many related works [33–35], the hierarchical architecture is often used for complex communication and control challenges for large unmanned systems. In this paper, we use a two-layer simplified model to describe the surveillance system.

**Figure 2:**
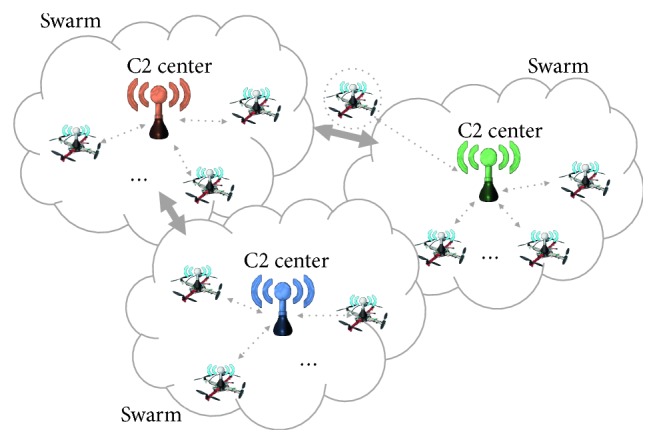
Cluster scale model.

As shown in Figure 2, the UAV surveillance model is divided into two parts: command and control center (C2 center) model and swarm model [36]. The C2 center is the control center of the swarm and can be summarized as “critical information exchange node where the control command is broadcast and the surveillance data is stored” [37]. A UAV swarm is a self-contained operation unit composed of sensing UAVs and can perform surveillance missions.

In the proposed model, the overall number of UAVs in the surveillance is set as *U*_sys_ and the UAV is regarded as a resource in the repository pool [34], where the total surveillance capacity is *P*_sys_.

Assume that we have *K*_sys_ ≥ 1, UAVs swarms, and each swarm has one C2 center. Each C2 center follows simple dynamics, given by the following equation:

(3) $$\begin{array}{c} \left\{\begin{array}{c} \dot{x}=v_{i}\;\;\cos\;\;u_{i},\\ \dot{y}=v_{i}\;\;\sin\;\;u_{i}, \end{array}\right. \end{array}$$

where (*x*_*i*_(*t*), *y*_*i*_(*t*)) ⊂ *E* is the position of the C2 center *i* at time *t*, *v*_*i*_ is the moving speed of the C2 center *i*, *u*_*i*_ is the heading angle of the C2 center *i*, and (*x*_*i*_(*t*_0_), *y*_*i*_(*t*_0_)) is the initial position of the C2 center *i*.

During the persistent surveillance mission, the aim of UAV swarm is to minimize the uncertainty of the target space or minimize the Φ(*t*) value in this model. Each UAV in the swarm is equipped with a high-resolution camera (to detect the target space). The information collecting capacity *P*_*i*_ of the UAV swarm is proportional to its number *U*_*i*_:

(4) $$\begin{array}{c} P_{i}=\frac{U_{i}}{U_{\text{sys}}}P_{\text{sys}},\\ \overset{K_{\text{sys}}}{\underset{i=1}{\sum }}P_{i}=P_{\text{sys}}. \end{array}$$

Consider that each UAV swarm has its own workplace *V*_*i*_ ⊂ *E*. Then, the decrease rate of information reward *C*_*i*_ for grids in *V*_*i*_ is shown in the following equation:

(5) $$\begin{array}{c} C_{i}=\frac{P_{i}}{\sum_{i=1}^{L_{x}}\sum_{j=1}^{L_{y}}p_{ij}}\;\text{for}\;\;p_{ij}\subset V_{i}. \end{array}$$

### 3.3. Performance Metrics

The aim of the persistent surveillance can be defined as maintaining an updated picture of the situation with minimal uncertainty. Hence, in this paper, we use the remaining information reward value to measure the performance of the control strategy. Assume that *J*(*σ*) is the maximum value of sum for the remaining information reward value in the target space:

(6) $$\begin{array}{c} J\left(\sigma \right)=\text{max}\left\{\underset{t⟶+\infty }{\text{lim}}\left[\overset{L_{x}}{\underset{i=1}{\sum }}\overset{L_{y}}{\underset{j=1}{\sum }}\text{sup}\phi \left(p_{ij},t\right)\right]\right\}. \end{array}$$

The task of the robot network is to estimate the state of the environment with minimal uncertainty. This can be achieved by minimizing *J*(*σ*). Assume that we use a strategy based on *σ*, then an optimal method is described by the following equation:

(7) $$\begin{array}{c} \sigma^{*}=\arg\;\;\min J\left(\sigma \right). \end{array}$$

## 4. Clustering-Based DTM Cooperative Persistent Scheme

In nature, herbivores can spontaneously develop clustered foraging behavior. The emerging process can be divided into three steps: turf updating, swarm reconfiguration, and individual feeding. Based on the DTM method, this section proposes a decomposition-based cluster-based persistent surveillance control scheme.

### 4.1. Multi-UAV Control Framework Using DTM

A persistent surveillance mission in a wide time-sensible environment has proved to be an NP hard problem even for a single UAV. This appears to be much more difficult when swarming UAVs are taken into account. To compound the problem, this study considers a situation wherein multiple UAV swarms conduct persistent surveillance missions synchronously in a time-sensible environment. Moreover, the workload balance and rapid instant-order response are requested to fulfill the task. An effective solution to such a problem is to divide the target space into several subregions and assign the number of UAVs in the subregion accordingly. Then, the complex search problem is simplified into a task allocation problem. In the swarm level, we only need to focus on the control strategy of the UAVs in the subregion, and communication and workload constraints no longer exist. Moreover, collisions between UAV subswarms are avoided. However, traditional methods lack flexibility and self-adaptability when confronted with persistent tasks with changing environments and orders. Consequently, when the orders of the mission change, the reconfiguration of the UAV swarm is difficult and the subswarm of UAVs might be trapped in an area with a local high repay value.

**Figure 3:**
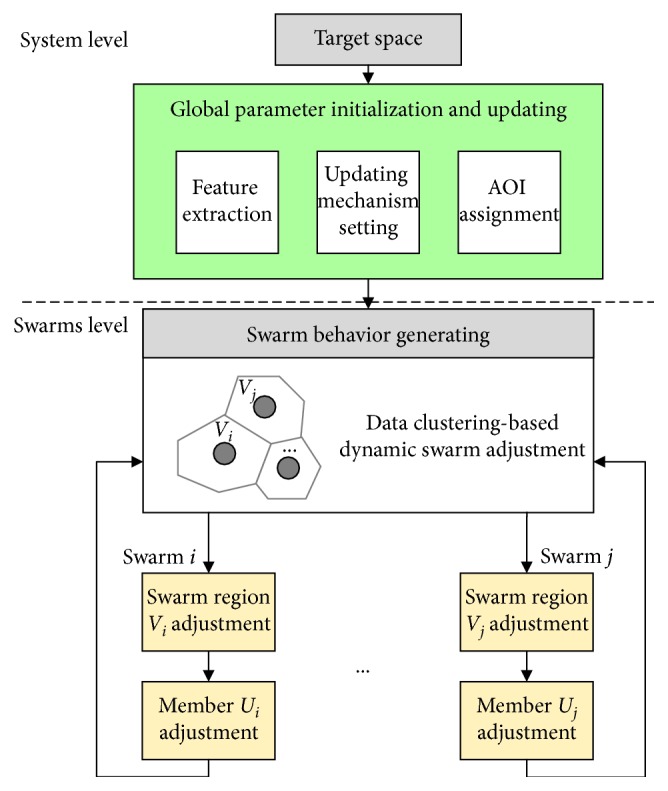
Hierarchy control framework.

In this paper, a hierarchy control framework using the DTM and a clustering algorithm are proposed to solve the persistent problem, as shown in Figure 3. The DTM control framework consists of two layers: centralized planning layer and distributed coordinating layer.

#### 4.1.1. System-Level Centralized Planning Layer

The centralized planning of the UAV swarm is conducted through the manipulation of the digital turf. Firstly, the searching space *E* is decomposed, and the DTM is used to approximate the searching payback value. The prior knowledge of the searching space is preloaded into the initial value of the parameter of the model. Then, the changes in the order and searching space are embodied throughout the updates of the AOI map, mission heat map, and difficulty capacity map of the digital turf. Through the iteration of the DTM, the changes in the target and mission intention are combined.

#### 4.1.2. Swarm-Level Distributed Coordination Layer

The distributed coordination of UAVs is carried out by the cooperation of C2 nodes of the subswarm. Specifically, in the process of swarm persistent surveillance, with the continuous changes in the environment, the C2 nodes of each UAV coordinate with each other and adjust their own spatial location, scope of jurisdiction, and jurisdiction subswarm size. The value of digital turf plants used to quantify the persistent surveillance task can be regarded as a weighted data point. In this paper, the subregion is divided according to the improved clustering algorithm (see Section 4.3.), and the cluster alerting node motion algorithm is designed to make the allegation node spontaneously move to the weighted percussion of the whole reconnaissance subregion.

### 4.2. Representation of Uncertainty: The Digital Turf Model

#### 4.2.1. Concept of Digital Turf Map

The digital turf model (DTM) is inspired by several factors: (1) the law of nature plant growth and reproduction, (2) routes and cluster behavior of herbivores, and (3) a reconfigurable distributed artificial intelligence system and the concept of regarding the whole swarm as a resource pool in the cloud.

**Figure 4:**
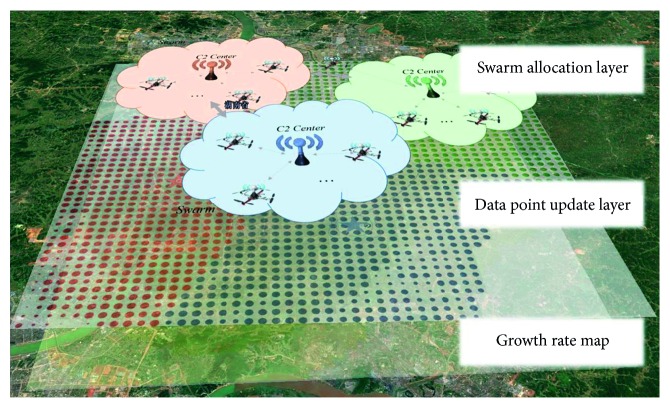
DTM map.

As shown in Figure 4, the basis of the DTM is the use of the quantity of the digital turf to represent unknown information and the use of the renewable digital turf to describe the time-dependent field of unknown information in the mission environment of the persistent surveillance. With cluster intelligence, the surveillance swarm can adaptively reconfigure and move in the changing environment.

The DTM method predominantly consists of two processes: region decomposition and initialization, and updating.

#### 4.2.2. Field Initializing and Updating

In the initialization phase, the model is assigned certain attributes. Meanwhile, the prior knowledge of the mission and the environment is stored in the model as the initial values of the parameters.

To set up the model, a mixed information map of DTM is constructed. The first step is the decomposition and rasterization of the mission area. As shown in Figure 1, the mission region is discretized into *L*_*x*_ × *L*_*y*_ grids, where the size of the grid is set to be equal to the field of view (FOV) of the UAVs. Each grid is associated with its digital turf value Φ(*t*), representing the information value in this grid.

In the DTM, parameters *r*(*p*_*ij*_, *t*) and *k*(*p*_*ij*_, *t*) are used to describe the growth rate and maximum value of the DTM grid:

(8) $$\begin{array}{c} R=\left[\begin{array}{ccc} r\left(p_{11},t\right) & r\left(p_{12},t\right) & \cdots \\ r\left(p_{21},t\right) & r\left(p_{22},t\right) & \\ \vdots & & \ddots \end{array}\right],\\ K=\left[\begin{array}{ccc} k\left(p_{11},t\right) & k\left(p_{12},t\right) & \cdots \\ k\left(p_{21},t\right) & k\left(p_{22},t\right) & \\ \vdots & & \ddots \end{array}\right]. \end{array}$$

During the surveillance process, the AOI of the mission changes. The fertile switch parameter of DTM *s*(*p*_*ij*_, *t*) determines whether a grid is inside the AOI at a given time. The switch matrix *S* at a given time is described as follows:

(9) $$\begin{array}{c} S\left(t\right)=\left[\begin{array}{ccc} s\left(p_{11},t\right) & s\left(p_{12},t\right) & \cdots \\ s\left(p_{21},t\right) & s\left(p_{22},t\right) & \\ \vdots & & \ddots \end{array}\right], \end{array}$$

while

(10) $$\begin{array}{c} s\left(p_{ij},t\right)=\left\{\begin{array}{cc} 0, & p_{ij}\;\subseteq \text{ AOI},\\ 1, & p_{ij}\;⊄\text{ AOI}. \end{array}\right. \end{array}$$

Given *N*_AOI_(*t*) as the number of AOI grids in the whole environment, we have

(11) $$\begin{array}{c} \overset{W}{\underset{i=1}{\sum }}\overset{L}{\underset{j=1}{\sum }}s\left(p_{ij},t\right)=N_{\text{AOI}}\left(t\right). \end{array}$$

Assuming the initial value of the digital turf in each grid is *ϕ*(*p*_*ij*_, *t*_0_), the initial DTM field can be initialized considering the following AOI factor:

(12) $$\begin{array}{c} \Phi_{t_{0}}=\Phi \left(t_{0}\right)\times S\left(t_{0}\right). \end{array}$$

In addition, while performing the persistent surveillance task, the DTM map updates continually as follows:

(13) $$\begin{array}{c} \dot{\phi }\left(q_{i},t\right)=\left\{\begin{array}{cc} r\left(q_{i},t\right)-c\left(q_{i},t\right), & q_{i}\in Q,\\ 0, & q_{i}\notin Q. \end{array}\right. \end{array}$$

### 4.3. Task Allocation of UAV Swarms Using the Clustering Algorithm

#### 4.3.1. Target Space Decomposition Using Data Cluster Methods

The original values estimated in the DTM map are not well divided into subregions for direct usage for UAV swarms, owing to several reasons. One is the large amount of data points, which is difficult for the command center to calculate. Another reason is that the AOI area of persistent surveillance mission changes dynamically over time. Such cases often occur in many WiSAR scenarios, such as earthquake rescue and search missions where occasional aftershocks occur.

Therefore, an appropriate computational method is required for the dynamic assignment of the target space. Such aggregation can be performed by different unsupervised competitive learning algorithms, such as a clustering algorithm and neural gas and its modification.

In this study, a weighted k-means clustering method is used to determine the reconnaissance subregion of the UAV subswarm. Each grid of the DTM is regarded as a data point, and the repay value associated with the grid is taken as the weight of the data point. Using the weighted k-means method (shown in Algorithm 1), we can decompose the searching space dynamically, and the C2 nodes are assigned to the geometrical center of the subregion.

#### 4.3.2. Swarm Moving and Resource Assignment Strategy

Note that in actual application, the swarm is limited by its dynamic and number constraints; the actual surveillance effect relies on the location and resource distribution of the swarms. This paper presents a control strategy by which the surveillance system moves the swarms and allocates surveillance resources using the approximate *φ* value of the DTM map.

To be more precise, the assignment of swarms is realized through the control of the location of the C2 center of each swarm (*x*_*i*_(*t*), *y*_*i*_(*t*)) and the number of UAVs attached to each swarm *U*_*i*_. As aforementioned, K-means clustering is a popular dynamic decomposition algorithm that can respond rapidly to change and also balance the workload. In this section, we take the advantages of the K-means-based algorithm and use a weighted function to drive the swarms to spread out over the task space while dynamically adjust their attached numbers.

**Figure 5:**
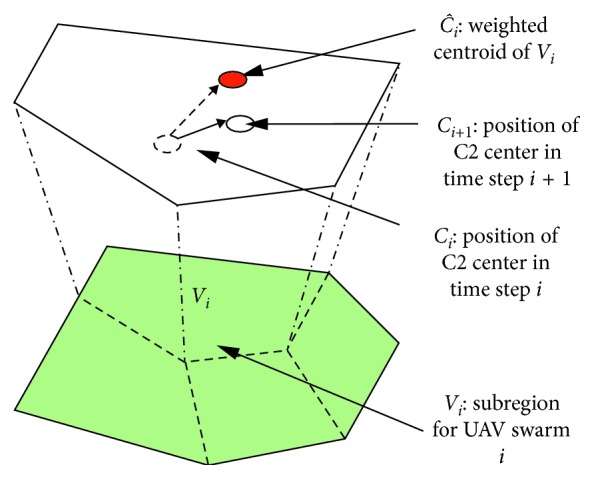
Moving process of C2 centers.

An overview of our strategy is shown in Figure 5.

## 5. Simulations

This section presents the numerical simulations to illustrate the efficiency and convergence of the proposed framework.

All the examples use the proposed cooperative control scheme, where the two-layer control strategy is performed via Algorithm 2; the updating of the spatiotemporal field via Algorithm 1; and the subregion and routine of each UAV swarm is generated via Algorithm 3. The searching area with a scale 5000 × 5000 m is taken as the target area, which is further decomposed into 50 × 50 grids. The initial locations of the C2 nodes are generated randomly. Unless otherwise specified, the values of the remaining required parameters are set as follows: number of UAV swarms Nu = 3, total time *T* = 500, FOV radius Rs = 100 m, maximum capacity of information in one grid *K* = 100, and number of area of interests *N*_AOI_ = 2000.

### 5.1. Persistent Surveillance by DTM

**Figure 6:**
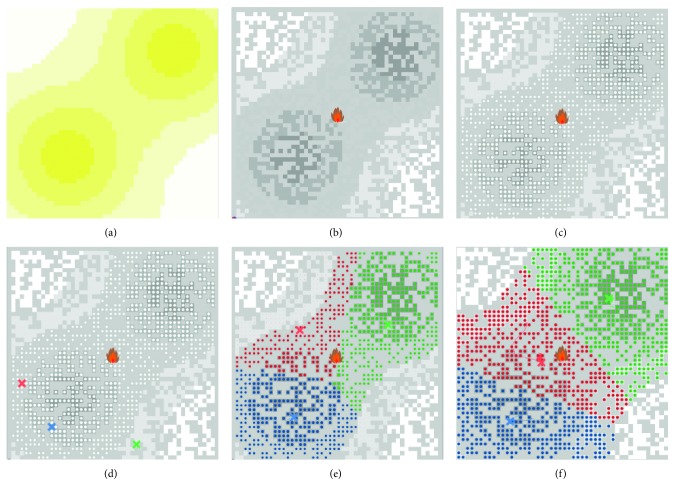
Dynamic swarm adjustment results of the proposed control scheme. (a) Growth rate map of DTM field; (b) AOI map of DTM field; (c) data points in DTM field; (d) initial location of C2 centers for three-swarm system; (e, f) dynamic adjustment of multiswarm system.

Consider a three-swarm persistent surveillance mission executed over the target area in Scenario I, as shown in Figure 6. The initial location of the command and control center of each swarm is randomly chosen in the target area. The growth rate of the data points in each grid is shown in the heat map in Figure 6(a) (darker color represents higher growth rate).

As shown in Figure 6(d), according to the proposed framework, the C2 center of each UAV swarm adjusts its location and task domain as the environment continues to change. And the changing process of their domain is shown in Figures 6(e) and 6(f).

As shown in Figure 6(c), as the environment of the persistent task changes, the deployment of the C2 centers of the UAV swarms changes accordingly, and all the data points in AOI are included in the subregions of the UAV swarms. Meanwhile, the areas with the higher growth rate are prioritized. The surveillance system shows that the UAV swarms can adjust their geographical distribution and size of domain to adjust to changes in the environment. At the same time, when the surveillance system stabilizes, the final distances between the C2 centers are (150, 900) m, (500, 400) m, and (1500, 200) m, respectively; this indicates that there is no overlapping or conflicting situations.

### 5.2. System Reconfiguration in Event-Driven Scenario

In this section, we examine the simulated response of the system framework proposed by the AOI based on the environment. We are particularly concerned about the reconnaissance system in the AOI escort, movement, and expansion mode of adjustment; in this case, the DTM turf AOI matrix is adjusted constantly.

#### 5.2.1. Changing AOI Mode Setup

To better test our model in changing AOI, we designed three changing modes in the environment: escort mode, retractable mode, and “wildfire” mode.

In the escort mode, the UAV swarms follow the target object to conduct a guarding and protection mission. The AOI is set around the target region center point P, forming a fixed radius Rd of reconnaissance area and reconnaissance area with the changes in center point P coordinates (*x*_p_, *y*_p_).

In the expansion mode, the coordinates of the center point P of the AOI remain constant while the radius of AOI changes over time.

In the “wildfire” mode, the reconnaissance area takes different “fire” coordinates as the center, forming the reconnaissance area, respectively.

#### 5.2.2. System Performance in Different Modes

**Figure 7:**
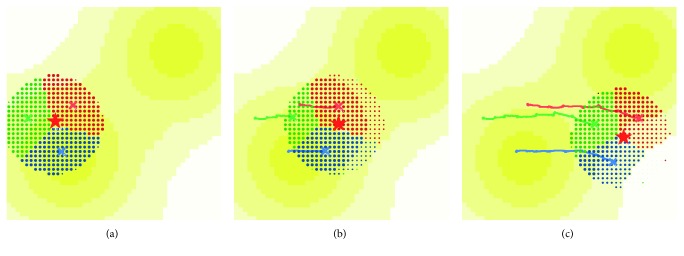
Dynamic swarm adjustment results in escort mode. (a) Initial swarm locations; (b) swarm movement in moving AOI; (c) final deployment of UAV system.

Figures 7(a)–7(c) shows the movement of the UAV swarms in the escort mode. It can be seen that the movement of the AOI and the allegations of the unmanned aerial vehicle clusters can spontaneously follow their movements and keep tracking the target. Meanwhile, the workload balance of the whole system is obtained as well.

**Figure 8:**
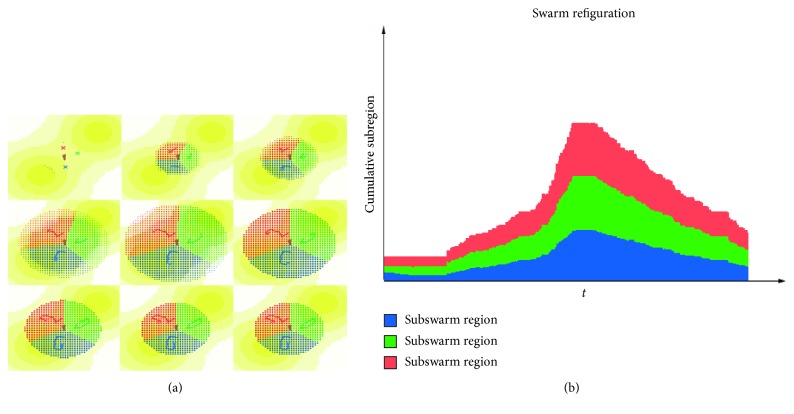
Example of dynamic swarm adjustment results in expansion mode. (a) Swarm adjustment in expansion mode and (b) the reconfiguration of swarm domain scale.

Figure 8 shows the UAV cluster changes with the AOI of the DTM in the expansion mode. In addition, as the scope of the reconnaissance changes, the subregions of each UAV swarm changes simultaneously.

**Figure 9:**
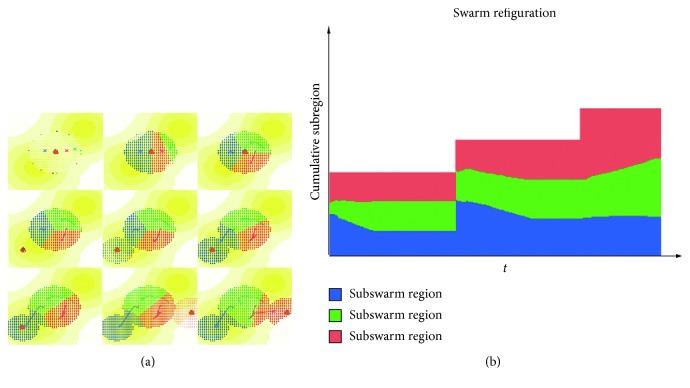
Example of dynamic swarm adjustment results in emergency mode. (a) Swarm adjustment in expansion mode and (b) the reconfiguration of swarm domain scale.

Figure 9 shows the dynamic adjustment process of the swarms in the emergency mode. In the emergency mode, new events occur unexpectedly. It can be seen in the figure that the swarms can respond in time and cover the new area.

### 5.3. Comparison between Different Methods

To further evaluate the proposed control scheme, traditional methods, including random search and zigzag search, are used for comparison. In the random mode, we assume each UAV executes its surveillance task independently. As the UAVs in the random mode cannot distinguish the AOI, the surveillance force of the whole system is allocated randomly in the task environment E. The zigzag search method is widely used for its simplicity and high efficiency. In this section, we divide the task environment into three equal subregions, each with a swarm of UAVs.

**Figure 10:**
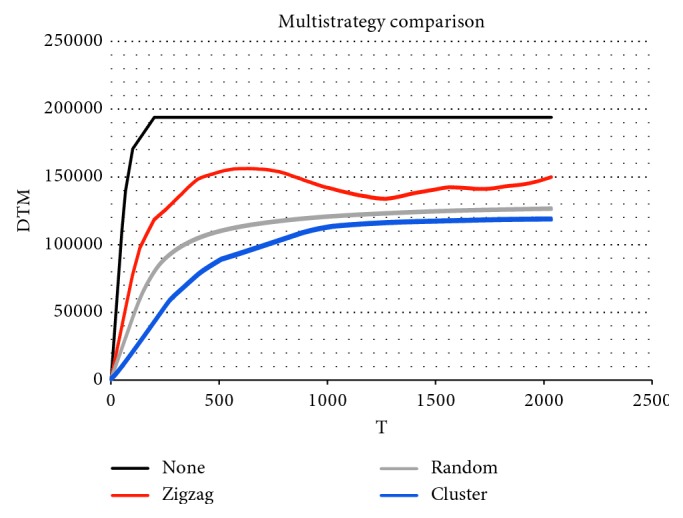
Real-time simulation of different methods.

For brevity, only the case with no unpredictable emergency is discussed in this section. To compare these methods, we evaluate the remaining value of likelihood function Φ(*t*) of the DTM field. The real-time simulation of likelihood value Φ(*t*) is shown in Figure 10. The thick black line represents the DTM field value of the no-surveillance trajectory Φ_null_(*t*). From the figure, we can see that that the zigzag trajectory has a bounded efficiency. Moreover, the figure shows that our proposed control strategy outperforms other methods in the simulation.

## 6. Conclusions

This paper presents the digital turf model (DTM) as a distributed control method for multi-UAV persistent surveillance and self-reconfiguration. In this paper, we proposed a two-layer control scheme for a multiswarm UAV persistent surveillance task. The paper argued that self-reconfigurable UAV swarms demand a new methodology for organization and cooperation, and the biological concept of herbivore swarm behavior in grasslands provides inspiration. First, we proposed the DTM map as a distributed scalar field to estimate the information reward value of the spatiotemporal environment. Second, using the DTM, we presented a control scheme that allows the problems of area decomposition, partition, and coverage in a multi-UAV remote sensing context to be addressed in a common framework. The advantage of the DTM and the data clustering-based control scheme includes its locality, simplicity, robustness, and self-reconfiguration.

Simulations have shown that this DTM approach can support many unique features of self-reconfigurable UAV swarms, including adaptive location adjustment in dynamic situations, decentralized and distributed control for collaboration between autonomous modules, on-line reconfiguration, and scalability to larger and multiple robotic systems. In future work, we will consider using the complex network to better model the on-line behavior of UAV swarms, and other data-based algorithms will be tested to generate the optimal path for UAV swarms.

## Figures and Tables

**Algorithm 1:**
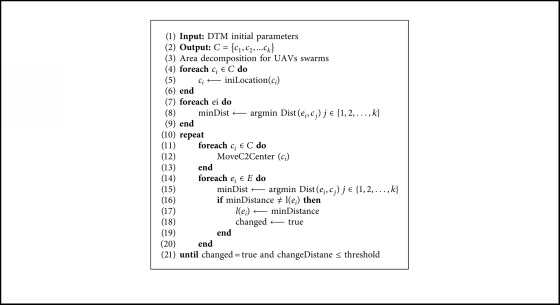
Grid-based weighted k-means algorithm.

**Algorithm 2:**
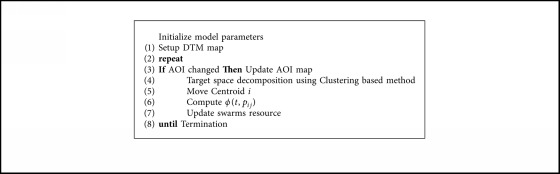
Overview of multiswarm persistent control framework.

**Algorithm 3:**
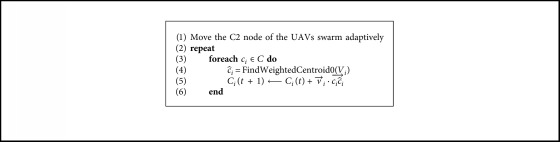
C2 center move algorithm.

## Data Availability

The data used to support the findings of this study are included within the article.

## Glossery

*p*_*ij*_: Position of grid *i*
*E*: Task space of mission
*qt*_*i*_: Data point *i* at time *t*
*Q*(*t*): Area of interest at time *t*
*ϕ*(*q*, *t*): Scalar field value at point *q* at time *t*
*Φ*(*t*): Spatiotemporal field (likelihood function)
*U*_*i*_: Number of UAVs in swarm *i*
*U*_sys_: Number of UAVs in system
*P*_*i*_: Task execution ability of swarm *i*
*P*_sys_: Task execution ability
*V*_*i*_: Swarm domain for swarm *i*
*C*_*i*_: Decrease rate of *ϕ* (*q*, *t*) in *V*_*i*_
*σ*^*∗*^: Swarm deployment trajectory
*J*(*σ*): Maximum sum of the remaining information reward value.
